# Validating Trend-Based End Points for Neuroprotection Trials in Glaucoma

**DOI:** 10.1167/tvst.12.10.20

**Published:** 2023-10-31

**Authors:** Giovanni Montesano, David F. Garway-Heath, Alessandro Rabiolo, Carlos Gustavo De Moraes, Giovanni Ometto, David P. Crabb

**Affiliations:** 1 City, University of London, Optometry and Visual Sciences, London, UK; 2 NIHR Biomedical Research Centre, Moorfields Eye Hospital NHS Foundation Trust and UCL Institute of Ophthalmology, London, UK; 3 Department of Health Sciences, Università del Piemonte Orientale “A. Avogadro,” Novara, Italy; 4 Eye Clinic, University Hospital Maggiore della Carità, Novara, Italy; 5 Bernard and Shirlee Brown Glaucoma Research Laboratory, Edward S. Harkness Eye Institute, Columbia University Irving Medical Center, New York, NY, USA

**Keywords:** visual field (VF), glaucoma, neuroprotection, clinical trials

## Abstract

**Purpose:**

The purpose of this study was to evaluate the power of trend-based visual field (VF) progression end points against long-term development of event-based end points accepted by the US Food and Drug Administration (FDA).

**Methods:**

One eye from 3352 patients with ≥10 24-2 VFs (median = 11 years) follow-up were analyzed. Two FDA-compatible criteria were applied to these series to label “true-progressed” eyes: ≥5 locations changing from baseline by more than 7 dB (FDA-7) or by more than the expected test-retest variability (GPA-like) in 2 consecutive tests. Observed rates of progression (RoP) were used to simulate trial-like series (2 years) randomly assigned (1000 times) to a “placebo” or a “treatment” arm. We simulated neuroprotective “treatment” effects by changing the proportion of “true progressed” eyes in the two arms. Two trend-based methods for mean deviation (MD) were assessed: (1) linear mixed model (LMM), testing average difference in RoP between the two arms, and (2) time-to-progression (TTP), calculated by linear regression as time needed for MD to decline by predefined cutoffs from baseline. Power curves with 95% confidence intervals were calculated for trend and event-based methods on the simulated series.

**Results:**

The FDA-7 and GPA-like progression was achieved by 45% and 55% of the eyes in the clinical database. LMM and TTP had similar power, significantly superior to the event-based methods, none of which reached 80% power. All methods had a 5% false-positive rate.

**Conclusions:**

The trend-based methods can efficiently detect treatment effects defined by long-term FDA-compatible progression.

**Translational Relevance:**

The assessment of the power of trend-based methods to detect clinically relevant progression end points.

## Introduction

When managing glaucoma, visual field (VF) damage is quantified and monitored with Standard Automated Perimetry (SAP). Moreover, SAP is a key outcome measure in randomized clinical trials (RCTs) for glaucoma. It is also one approved by the US Food and Drug Administration (FDA)^1^ and other regulatory bodies for glaucoma because it is a direct measure of visual function and is related to changes in vision-related quality of life (QoL).^2^^–^^6^ However, criteria defining progression as described by regulatory authorities are somewhat vague, and, as yet, there is no published consensus from the clinical research community. Previous RCTs have used various forms of event analysis, most recently based on the algorithm implemented in the Guided Progression Analysis (GPA; previously Glaucoma Change Probability) in the Humphrey Field Analyzer (Zeiss Meditec, Dublin, CA, USA). This method was inherited from the Early Manifest Glaucoma Trial and identifies changes from baseline, exceeding the limits of test variability derived from a test-retest cohort.^7^^,^^8^ It has been reported that the FDA currently accepts significant changes in at least five VF locations in two consecutive visits as a valid outcome.^1^ Besides the GPA criteria, the FDA has also indicated that a change in sensitivity of 7 dB or more from baseline is acceptable to determine change.^1^ This latter criterion has been used in previous publications^9^ and recently in a gene therapy trial for retinitis pigmentosa looking at VF improvement.^10^ Thresholds for change based on a simple number have the advantage of not requiring normative variability limits for a specific device.

An alternative approach to determine progression is to use the trend of VF change,^11^^–^^15^ usually as a linear function of time. This has the advantage of quantifying the speed of progression rather than a progression event. For RCTs, this method has been shown, through simulations, to be much more powerful than event-based analyses in detecting treatment effects.^12^ Importantly, effect sizes are likely to be small for any add-on treatment beyond intraocular pressure (IOP) lowering and this is particularly relevant for neuroprotection trials, where VF outcomes are mandated, requiring long follow-ups and a prohibitively large number of patients to be sufficiently powered.^16^^–^^18^ Moreover, the rate of progression (RoP) calculated through trend analyses over 2 years has been recently shown to be predictive of FDA-consistent outcomes over a 5-year period in a clinical cohort.^19^^,^^20^ This has led the scientific community and the industry to push for acceptance of trend-based outcomes to improve the feasibility and efficiency of RCTs.^12^^,^^17^^,^^18^

The optimal methodology to compare RoPs between arms of an RCT is not yet established. Measurements of the difference in the average RoP with linear mixed models (LMMs), as a standard tool for longitudinal measurements, has gained traction in recent years.^11^^,^^12^^,^^14^ However, the power of this method to detect future development of FDA-compatible outcomes is still untested. Moreover, where an event-based analysis estimates a change in the risk of developing a progression event for individual patients and quantifies how many of them are likely to benefit from a treatment, an average difference in RoP from LMM does not have such a straightforward interpretation. In previous publications^21^^,^^22^ we have shown the potential of trend-based analyses to produce continuous estimates of the time to change by a prespecified threshold. This approach proved particularly useful for the 5-year VF results of the HORIZON trial,^22^ where it was able to highlight that a significant difference in the average RoP detected with LMMs was actually driven by a subset of fast progressors in one arm. This distinction dramatically changed the interpretation of the results.

In this work, we use data from a large dataset from 3352 individuals with series of at least 10 VF tests to detect patients that would develop progression according to FDA-compatible criteria. We then use simulations to systematically calculate the power of different trend-based methods in detecting various simulated neuroprotective effects to reduce the risk of long-term FDA-compatible progression.

## Methods

### Database

The characteristics of this database have been described in detail elsewhere.^23^^–^^25^ VF data were extracted from the electronic medical records (EMRs; Medisoft; Medisoft Ltd., Leeds, UK) from 5 National Health Service Hospital Trust glaucoma clinics in England in November 2015. In short, all patient data were anonymized at the point of data extraction and subsequently transferred to a single secure database at City, University of London. Subsequent analyses of the data were approved by a research ethics committee of City, University of London. The study adhered to the Declaration of Helsinki and the General Data Protection Regulation of the European Union. All VFs were 24-2 tests performed with a Humphrey Field Analyzer (HFA), Goldmann III stimulus, and the Swedish Interactive Testing Algorithm (SITA Standard or SITA Fast). The database included 576,615 VFs from 71,361 patients recorded between April 2000 and March 2015. We excluded VFs with a percentage of false positive errors ≥ 15%. No exclusion criteria were applied on fixation losses or false negative errors.^26^ We selected all patients with at least 10 VFs recorded over at least 4 years in one or both eyes and a mean deviation (MD) worse than −2 dB in at least 2 (not necessarily consecutive) VFs in the same eye, similarly to previous studies.^27^^–^^29^ This was to overcome the lack of a definitive label indicating a diagnosis of glaucoma. However, it is reasonable to assume subjects with this level of VF loss and frequency of VF monitoring in a glaucoma clinic were likely to be either strong glaucoma suspects or persons with glaucomatous optic neuropathy. However, it should be kept in mind that other diseases might have caused VF loss, such as neurological or vascular events. Although these were partially filtered out by using diagnostic labels from the EMR, they might still be included in the dataset. VFs performed after any ocular surgery other than cataract extraction were also excluded. Finally, only one eye from each patient was selected, at random if both were eligible. The final selection included 44,371 VFs from 3352 eyes.

Patients’ demographics were (median [interquartile range]): age 68 [60, 75] years; best corrected visual acuity (BCVA) 0 [−0.1 to 0.2] logMAR; average IOP 16 [14, 18] mm Hg; average MD −6.44 [−11.06 to −4.07] dB; and average pattern standard deviation (PSD) 5.68 [3.27, 9.06] dB. Average values were calculated over all the available measurements within the time frame of the VF tests. The median length of follow-up was 11 [8, 13] years and the number of VFs per series was 12 [11, 15].

### Simulated Trials and Power Calculations

**Figure 1.**
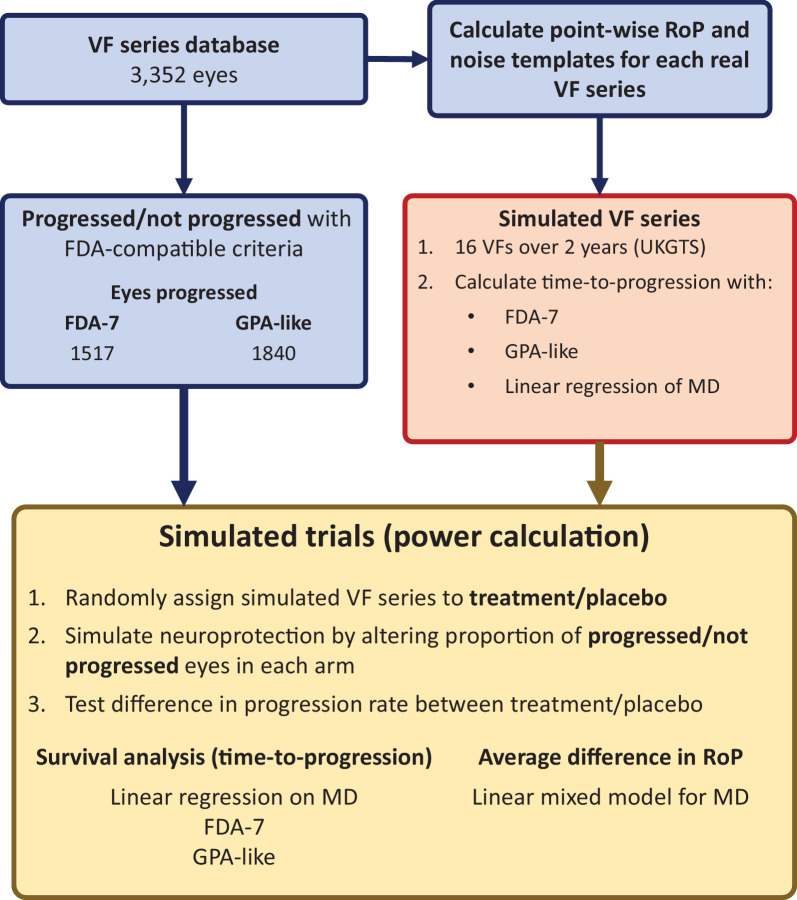
Flow-chart of the methods. All eyes are classified as progressed or not progressed based on either the FDA-7 or the GPA-like criteria (see text) on their real visual field (VF) series. The real VF series are also used to calculate the best estimate of the point-wise rate of progression (RoP) and the noise templates. These are used to simulate one VF series for each eye according to the United Kingdom Glaucoma Treatment Study (UKGTS) scheme. The time-to-progression for each simulated series is calculated using change from baseline according to the linear regression of mean deviation (MD) over time, the FDA-7 and the GPA-like criteria. Trials are then simulated by randomly allocating simulated VF series from progressed and not progressed eyes (according to the real series) to a placebo or treatment arm (1000 simulations for 13 sample sizes for 4 neuroprotective effects). Neuroprotection is simulated by altering the proportion of progressed/not progressed eyes in the two arms. The difference between the two arms is then estimated by survival analysis (for time-to-progression) or by linear mixed modeling (for the average difference in the RoP of MD over time).

The methodology for the simulated trials and the power calculations is detailed below and reported in the flowchart in Figure 1.

### Detection of FDA-Compatible Progression

For our primary analysis, a progression event was a negative change in sensitivity from baseline of 7 dB or more at the same location, in at least 5 locations in 2 consecutive VFs in the whole VF series available in the dataset (FDA-7 criterion). The baseline was calculated as the average of the first two tests in the series. The date for the event was that of the first of the two consecutive tests where the change was identified.

A secondary analysis was also conducted by defining a progression event according to GPA-like criteria. This aimed at identifying progressing locations as those progressing more than the lower 5% limits of test-retest variability for a given baseline pattern deviation (PD) value.^7^^,^^8^^,^^30^ The lower 5% test-retest limits was estimated from a test-retest dataset freely available with the *visualFields* package^31^ for R (R Foundation for Statistical Computing, Vienna, Austria), composed of 12 tests performed over a period of 12 weeks in 1 eye of 30 patients with glaucoma.^32^ The PD for each VF was calculated with the use of dedicated functions in the *visualFields* package using an internal normative database.^31^ Baseline PD values were calculated using the average of two VFs. All possible combinations of two tests were used as baseline and associated with all possible retest values from the remaining 10 tests. The lower 5% limit was calculated for each rounded sensitivity. The PD values were then calculated for each VF in the clinical dataset and test-retest limits for each location were established based on its baseline PD value (average of the first 2 fields). Progressed eyes were then detected as those showing a change in the same five or more locations in two consecutive VFs, similarly to the memantine neuroprotection trial.^33^

### Simulation of Visual Field Tests

VF tests for the simulated trials were generated using a method similar by that described by Wu and Medeiros.^34^ In brief, the point-wise slopes and intercepts were estimated for each eye with a hierarchical model grouping observations over time by location and each location by VF clusters according to Garway-Heath et al.^35^ The method also accounted for censoring at the floor level (0 dB) to avoid bias of the slopes. The methodology for the hierarchical model is described in detail in a previous publication.^36^ These point-wise estimates were used to generate best available estimates (BAEs) of the “true” sensitivity values using the entire series in the clinical database. To model perimetric noise, each observed VF was transformed into a noise template by subtracting the BAE of sensitivity from the observed values (residuals). Then, each residual value was transformed into a cumulative probability value using the cumulative distribution function (*cdf*) of a Gaussian distribution with zero mean and standard deviation (SD) varying with the BAE of sensitivity according to equation 3 in table A2 in Montesano et al.^36^ This process generated noise templates that are normalized according to the expected variability for a given sensitivity.

For the simulations, the assumed “true” sensitivity values at each time point were generated from the hierarchical estimates of point-wise intercepts and slopes. Perimetric noise for each eye was then applied by choosing a template at random from those available for that specific eye. The probability values in the template were transformed back into residuals, using a Gaussian *cdf* with the SD determined, as previously explained according to the underlying “true” simulated point-wise sensitivity, and added to the simulated values. The templates modeled global fluctuations in performance which are known to be the major source of variability for global indices, such as the MD,^34^^,^^37^ and retained the specific variability of each subject.^16^^,^^34^

Following the United Kingdom Glaucoma Treatment Study (UKGTS) testing scheme,^16^^,^^38^ we simulated 16 tests over 2 years at 0, 2, 4, 7, 10, 13, 16, 18, 20, 22, and 24 months, with 2 clustered tests at 0, 2, 16, 18, and 24 months. For each simulated test, we calculated the PD numeric maps and the MD using dedicated functions in the *visualFields* package.^31^ The FDA-7 and GPA-like criterion were calculated for the simulated series as previously explained.

### Trend-Based Progression Methods

We compared two different methods of trend-based analysis for the simulated trials (see below). The first was an LMM modeling MD over time,^12^^,^^16^ with the eye as the random effect. Fixed effects were the time from baseline (in years) and the arm of the trial (0 for placebo and 1 for treatment). The interaction between the trial arm assignment and time modeled the average difference in slope between the two groups (outcome of interest for which significance was assessed). Random intercepts and slopes were used to model variability in baseline damage and RoP among different eyes.

**Figure 2.**
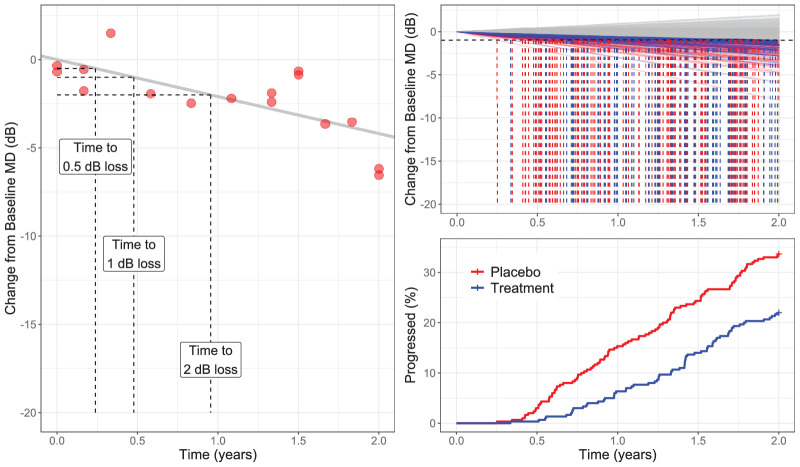
Example of how the trend-based time-to-progression is calculated for an individual subject (**A**) and on a clinical trial cohort (**B**). The horizontal dashed line indicates the threshold used to define the event. The trend lines for the eyes that reached the event are color-coded, whereas the others are shown in *gray*. The survival curves (**C**) are built based on the events marked as *vertical lines*. Note that the baseline (intercept) is set to zero for this analysis (i.e. only the slope is considered, because it represents the change from baseline over time). MD, mean deviation.

The second method consisted of calculating the RoP for each simulated series using Ordinary Least Squares (OLS) regression of MD over time. This RoP was then used to obtain a continuous estimate of the time taken for each eye to progress to a prespecified threshold from baseline. For example, a prespecified change of 1 dB below baseline would take 2 years for an eye progressing estimated to progress by 0.5 dB/year (see Fig. 2). This change was considered as an event in a Cox proportional-hazard model, testing the difference in survival time between the two arms of the trial. Eyes with positive slopes or with slopes that did not reach the prespecified threshold change for the event were not identified as progressing and were treated as censored observations at the time of the last VF. This method has been previously used for the VF analysis in TAGS.^21^ In the case of the TAGS trial, the global RoP was based on point-wise hierarchical model of sensitivity. Note, however, that the method can be used for any model, linear or nonlinear, able to produce a continuous estimate of VF sensitivity over time. It should be noted that individual slopes can also be extracted from an LMM using random effect estimates,^39^^,^^40^ but this method was avoided to prevent shrinkage to the grand mean and to maintain independence of the RoP estimates among different eyes. In this case, the OLS of MD was chosen for direct comparison with the results from the LMM. A schematic of the calculation is shown in Figure 2. Three threshold changes were chosen for this analysis, 0.5 dB, 1 dB, and 2 dB, which, over 2 years, correspond to a linear RoP of −0.25 dB/year, −0.5 dB/year, and −1 dB/year.

### Simulated Trial Experiments and Power Calculation

Eyes were labeled as progressed or not progressed according to whether they developed progression with either FDA-compatible criterion (FDA-7 or GPA-like) at any point in time in their clinical series of 10 or more VFs (i.e. the entire follow-up time). Each one of these eyes had one simulated series associated with it. The treatment effect was simulated by selectively sampling progressed and non-progressed eyes so that a different proportion of progressed eyes were allocated to the placebo and treatment arm. The placebo arm had the same proportion of progressed eyes as the overall clinical sample. The proportion for the treatment arm was instead diminished, according to the desired effect, quantified as a percent change in relative risk (RR). For example, in a sample of 100 eyes per arm with 50 of 100 progressed eyes sampled in the placebo arm, a 30% effect would be simulated by sampling 35 of 100 progressed eye in the treatment arm (RR = 0.7).

Because the likelihood of developing FDA-compatible events could be related to the level of damage, and because the level of damage has a known effect on perimetric variability,^41^^,^^42^ the sampling was stratified by baseline MD by dividing the patients into early (MD ≥ −6 dB), moderate (MD ≥ −12 dB), and fast (MD < −12 dB) This approach allows for the relative proportions of these groups in each arm to always be same as the overall sample (60%, 26%, and 14%, respectively). The baseline MD was calculated as the mean of the first two MD values in the clinical series.

We simulated 0% (no effect, false positive differences), 20%, 30%, and 50% effect. We simulated 1000 2-year trials for each effect size and for 13 sample sizes (from 100 to 1300 eyes per arm, every 100). The 2 methods of trend-based analysis (LMM and Cox model) were applied to each simulated trial sample and a *P* value < 0.05 was considered statistically significant. The three cut-offs for the survival analysis were all tested and reported separately. An additional result was the significance of the smallest of the three *P* values obtained with all the cut-offs (time-to-event, combined), corrected for three tests with the Bonferroni-Holm method.^43^ Finally, a survival analysis for FDA-compatible criteria on the simulated series was also performed for comparison.

Confidence intervals for the power was calculated as 1.96 × SE, where SE is the standard error for the probability of a binomial process, calculated as $SE=\sqrt{p_{p<0.05}*\left(1-p_{p<0.05}\right)/N}$, where *p*_*p* < 0.05_ is the proportion of significant *P* values in the simulations, and *N* is the total number of simulations. Note that these CIs are only meant to represent the precision of our estimates: statistical comparisons between power curves are meaningless in this context because the number of simulations can be increased arbitrarily to reach any level of significance.

## Results

**Figure 3.**
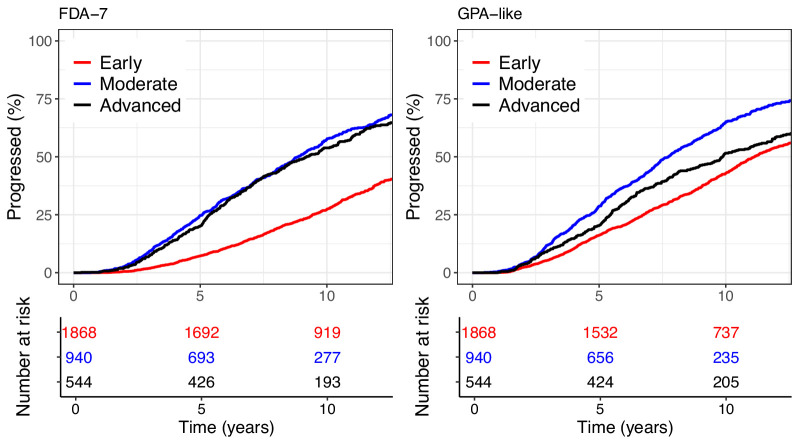
Survival curves using the two different FDA-compatible criteria used in this study applied to the clinical database, stratified by level of damage. Eyes with early, moderate, and advanced damage at baseline represented 60%, 26%, and 14% of the sample respectively. Censored data marks are omitted for clarity.

Forty-five and 55% of the eyes were labeled as progressed in the clinical database series with FDA-7 and GPA-like criteria, respectively. Figure 3 shows Kaplan-Meier curves according to the level of baseline damage. Such a high cumulative incidence of events is expected because of the relatively long follow-up time with, for example, a quarter of series having more than 13 years of follow-up. The Table reports some descriptive statistics for the patients in the progressed and not progressed groups according to the two criteria.

**Table.** Subjects’ Demographics for Progressed and Stable Eyes According to the Two FDA-Compatible Criteria Used in This Study

		Stable		Progressed		
		Mean (SD)	Median [IQR]	Mean (SD)	Median [IQR]	*P* Value
**FDA-7**	**Age** **,** **y**	63.86 (11.95)	65 [56, 73]	69.59 (10.14)	72 [64, 77]	<0.001
	**MD** **,** **dB**	−6.32 (5.09)	−4.43 [-6.94, −3.27]	−8.52 (5.23)	−7.02 [-11.25, −4.37]	<0.001
	**PSD** **,** **dB**	4.99 (3.34)	3.86 [2.35, 6.75]	7.15 (3.52)	6.87 [4.02, 9.96]	<0.001
	**IOP** **,** **mm** **Hg**	16.50 (3.23)	16.39 [14.37, 18.43]	15.50 (3.13)	15.31 [13.58, 17.28]	<0.001
	**BCVA** **,** **logMAR**	0.05 (0.25)	0.00 [-0.10, 0.20]	0.11 (0.28)	0.00 [−0.10, 0.20]	<0.001
	**Follow-up** **,** **y**	10.57 (2.80)	11.03 [8.45, 13.02]	11.08 (2.61)	11.60 [9.34, 13.25]	<0.001
**GPA-like**	**Age** **,** **y**	63.93 (12.10)	66 [57, 73]	68.53 (10.59)	71 [63, 76]	<0.001
	**MD** **,** **dB**	−6.32 (5.07)	−4.47 [−7.02, -3.27]	−8.13 (5.28)	−6.49 [−10.90, -4.04]	<0.001
	**PSD** **,** **dB**	5.04 (3.31)	3.95 [2.42, 6.85]	6.73 (3.62)	6.39 [3.47, 9.59]	<0.001
	**IOP** **,** **mm** **Hg**	16.32 (3.17)	16.18 [14.19, 18.28]	15.83 (3.25)	15.70 [13.81, 17.64]	<0.001
	**BCVA** **,** **logMAR**	0.05 (0.24)	0.00 [−0.10, 0.20]	0.10 (0.28)	0.00 [−0.10, 0.20]	<0.001
	**Follow-up, y**	10.58 (2.90)	11.06 [8.37, 13.12]	10.99 (2.56)	11.43 [9.18, 13.10]	0.001

MD, mean deviation; PSD, pattern standard deviation; IOP, average intraocular pressure; BCVA, average best corrected visual acuity; SD, standard deviation; IQR, interquartile range.

The *P* values were calculated with the Mann-Whitney Test.

**Figure 4.**
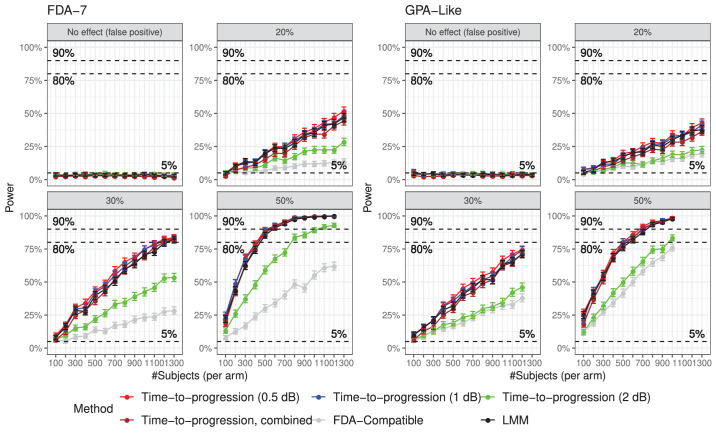
Power curves for different treatment effect sizes for all the proposed methods, using the two FDA-compatible criteria for classification (either GPA-like or FDA-7, according to which was used for the labelling of progressing eyes in the original clinical series). The error bars indicate the 95% confidence intervals.

The power curves for the different methods are reported in Figure 4. All methods detected a false difference in approximately 5% of the simulations, as expected. Eighty percent power was reached only by the trend-based methods for the 30% and 50% effect size within the maximum sample size achievable with our clinical cohort. Ninety percent power was only achieved for the 50% effect for the maximum sample size. Both of the FDA-compatible methods performed very poorly over the 2 years of the simulated trial, with a maximum power of only 62% for the FDA-7 and 76% for the GPA-like criterion with the largest effect. Most trend analyses performed very similarly, except the survival model for 2 dB threshold, which performed considerably worse than the LMM and the other cut-offs. The method combining all *P* values from the Cox models performed similarly to the LMM and the 0.5 dB and 1 dB thresholds. Numeric values are reported as Supplementary Tables.

## Discussion

In this analysis, we assessed the risk of reaching FDA-compatible VF end points over an average 11-year observation period in patients attending glaucoma clinics and used this as a ground truth for progression. We then modeled clinical trials with additional treatment effect sizes in the intervention arm of 20%, 30%, and 50% on the risk of reaching FDA-compatible end points and assessed the power of two trend-based methods to distinguish the treatment groups within a 2-year period (compared to the power of the FDA-compatible endpoints over the same period). We showed that, whereas the FDA-compatible methods themselves were not powerful enough to detect the effect within the 2-year span of the trial, most trend-based methods reached sufficient power for the largest effect sizes. This was despite the FDA-compatible criterion for the simulated trials being the same used for the identification of progressed eyes over the whole follow-up period.

Our results provide additional evidence that trend-based analyses appear to constitute a valid and more efficient alternative for the detection of treatment effects able to modify the long-term risk of VF progression, defined according to FDA-compatible criteria. This is especially important for the development and approval of novel treatments for patients with glaucoma without requiring RCTs with impossibly long durations and unrealistically large sample sizes. It is important to note that many seminal glaucoma RCTs managed to use event-based methods to show a significant effect for their proposed treatment with acceptable sample sizes and a relatively short follow-up time. However, the expected treatment effect size in these trials was large because they were comparing either medical treatment to no treatment/placebo^38^^,^^44^ or surgical intervention to laser treatment.^45^ In contrast, the effect expected from novel neuroprotective treatments is likely to be small in magnitude, especially because the placebo arm would still be treated according to the standard of care. However, even a small treatment effect on the RoP of VF damage is expected to prevent loss of vision in thousands of patients worldwide.^17^ Previous work in this field has shown trend-based analyses, and specifically LMMs, to be much more powerful than event-based analyses for the range of effects expected in a neuroprotection trial.^12^ Other strategies have been proposed to further improve power, such as the preferential recruitment of patients with lower test-retest variability^16^ or by enriching the sample with patients that are more likely to show VF progression.^9^

One unanswered question is how outcomes from trend-based analyses relate to VF progression outcomes compatible with the definition from regulatory bodies. For example, the FDA has not clearly stated what constitutes acceptable evidence of progression. However, in a consensus meeting,^1^ the methodology adopted in the memantine neuroprotection trial,^33^ akin to our GPA-like analysis, was deemed sufficient to provide evidence of VF change. This method identifies deterioration of individual locations from baseline as a change exceeding the lower 5% limit of the expected test-retest variability.^7^^,^^8^^,^^30^ Five or more consistent locations were required to show deterioration in two consecutive VF tests to identify a progression event in the memantine trial.^33^ Alternatively, a change of 7 dB from baseline has also been mentioned as evidence of a clinically meaningful difference.^1^ Although the description of this latter criterion is unclear, a 7 dB change at 5 or more locations has been adopted in a recent gene-therapy trial for retinitis pigmentosa, after communication with the FDA, to show improvement in visual function.^10^ However, none of the methods are expected to provide sufficient power to detect small effects owing to their poor sensitivity. It should also be noted that, despite these recommendations from the FDA, none of the landmark papers mentioned above used these specific criteria for progression.^38^^,^^44^^–^^46^ A recent paper by Medeiros and Jammal,^19^ has shown that the rate of MD progression over 2 years was predictive of FDA-compatible progression outcomes in the longer term. This was then confirmed by De Moraes et al.^20^ One limitation of those studies was the relatively small sample size and relatively short follow-up. In our work, we had access to a much larger pool of patients with follow-ups extending to 13 years (third quartile). Moreover, differently from previous work,^19^^,^^20^ we use these data to calculate the power of detecting an effect on FDA-compatible outcomes rather than simply showing association.

The most important aspect of our analysis is the comparison of different methods to detect trend-based progression. The standard LMM approach has been proposed to assess a difference in the mean RoP between the two arms of a trial.^12^^,^^16^^,^^40^ LMMs can be used to model the progression of global indices, such as MD, or point-wise sensitivity, with essentially the same interpretation of the results (average progression rate). One alternative we explored in our work is the use of trend-based estimates of progression for individual eyes (for example, but not necessarily, from OLS regression of the MD) to obtain a continuous estimate of the time for VF to change beyond a certain threshold from baseline. The time to progression can then be analyzed in the context of a type of survival analysis. We used this method in two recent publications testing the difference between the two arms of RCTs.^21^^,^^22^ In the simulations in the present work, we found no appreciable difference in power between the LMM and the time-to-progression approach, except for the 2 dB threshold (fast progressors). The lack of power for this extreme threshold is easily explained by the relatively small proportion of fast progressors in the overall sample. The power for this threshold was further reduced when progressed patients were analyzed with the GPA-like criterion. This is expected because fast progressing patients are more likely to have more advanced damage at baseline, which limits the ability of the GPA-like method to detect progression.^47^ One important aspect to consider is that neuroprotection RCTs might preferentially recruit patients with moderate or advanced disease. This would affect the statistical power of the various methods, and of the GPA-like event-based analysis in particular. Although the lower number of patients with moderate or advanced disease prevent us from producing power curves for each subgroup, we repeated our experiment excluding patients with early baseline damage (Supplementary Material). Interestingly, this improved the power curves for the trend-based methods and produced very similar results with the event-based methods. This is likely due to the fact that patients with moderate disease were more likely to progress. However, it should be kept in mind that our experiments cannot be an accurate test for the performance of the event-based methods, because the same event-based criteria were used to define “true” progression. This means that some limitations of event-based methods in moderate and advanced glaucoma, mainly the inability to detect progression for locations below a certain threshold of damage, would also be reflected in the definition of the ground truth.

It should be noted that, even with the most sensitive method, the number of participants required to reach sufficient power remains very large. These estimates are roughly in agreement with a previous analyses based on similar methodology,^12^^,^^16^ but the estimates were not expected to match exactly. The biggest difference is that, in those analyses, the neuroprotective effect was simulated by changing the RoP of each eye, making assumptions about how the slopes would be affected by the treatment. Here, we did not make such assumptions, but simply used the RoP actually observed in the clinical data and relied on the association between the RoP and the FDA-compatible criteria to change the distribution of slopes in the two arms by selective sampling. Interestingly, this translated to a roughly similar proportional change in the average RoP between the two arms, as estimated by the LMM (Supplementary Material).

Despite these similarities in power between LMMs and time-to-progression approaches, it is useful to make some important observations on the difference in interpretation of the results for clinical trials. As previously mentioned, LMMs estimate a difference in the average RoP between the two arms of an RCT. Whereas this can be a sensible index of a difference, it might not be sufficient to describe the clinical impact for individual patients. For example, a significant change in mean RoP might be equivalently determined by a small change for most of the patients or a large effect on a small subset of fast progressing eyes. Although these can both be valuable outcomes, they have profoundly different implications for translation into clinical practice. In the first case, virtually all patients would benefit from the treatment, whereas in the second case only a small proportion of eyes would see a meaningful advantage. A clear example of this has been recently provided by the analysis of the results of the HORIZON trial^22^: LMMs identified a significant difference in the average RoP; however, the time-to-progression analysis showed that this average difference was due to a larger proportion of fast progressors in one arm, with little to no change for the majority of patients. This dramatically changed the clinical interpretation of the results, with important consequences for the determination of cost effectiveness and development of treatment indications. Interestingly, for this specific trial, the most relevant cut-off was the most extreme (corresponding to > 1 dB/year loss), which was the least powerful in our simulations. There are also important consequences for the determination of a clinically meaningful effect. For example, defining a change in the average RoP by, say, 0.25 dB/year as a clinically significant outcome implicitly assumes that such an improvement will apply homogenously to the study population. In contrast, a trend analysis to calculate a time-to-progression can be directly used to define meaningful outcomes at the patient level and allows an interpretation and direct a quantification of the results in terms of risk reduction. In our analysis, we calculated the power for three different thresholds, 0.5 dB, 1 dB, and 2 dB. These correspond to a rate of progression >= 0.25 dB/year, 0.5 dB/year, and 1 dB/year. Naturally, the choice of an appropriate cut-off will need to be tailored to the different patients’ characteristics (such as age, stage of the disease, and comorbidities) and to the expected effect of the treatment. In our simulations, the 0.5 dB cut-off performed the best, but only marginally better than 1 dB, LMM, and the combination of multiple cut-offs. The latter uses multiple-test correction to combine the *P* values of all three cut-offs. Although this is not desirable for confirmatory RCTs, it may be a useful tool for exploratory analyses seeking to determine an expected treatment effect. It should be also noted that, although an equivalent comparison could be performed by testing the proportion of slopes more negative than a specific value, the use of survival analyses allows to account for censoring of patients with incomplete follow-ups, an inevitable feature of real RCTs, for example by limiting the detection of progression to the time of the last available VF test, avoiding extrapolation beyond the data. This approach was chosen for previous VF analyses.^21^^,^^22^

The need to choose a prespecified threshold of change might appear like a limitation of the time-to-progression method compared to LMM. However, this again derives from the difficulty of unambiguously defining a meaningful outcome with LMMs. In our simulations, similarly to previous literature, we considered a significant outcome as any with *P* value < 0.05, regardless of the magnitude of the difference. This is unlikely to be accepted by a regulatory body, such as the FDA. However, testing a prespecified difference, such as 0.25 dB/year, is prone to ambiguity: should this be interpreted as a result that is statistically significantly different from 0 dB/year and greater than 0.25 dB/year? What about a statistically significant difference whose 95% confidence intervals extend well below the predefined 0.25 dB/year cut off? Should the model instead test a significant difference from 0.25 dB/year? The time-to-progression analysis eliminates this ambiguity quantifying the reduction in risk of a well-defined progression outcome. An additional advantage of this method compared to more traditional event-based survival analyses is that it allows for higher temporal resolution even with sparse testing.^21^^,^^22^ Of course, the method currently assumes a linear model for VF progression. However, linear change of sensitivity has been shown to be an accurate and well accepted descriptor of VF progression,^40^^,^^48^^,^^49^ especially over a short period of time, such as the 2 years of this simulated trial. It should be pointed out, however, that the methodology can be extended to any model, linear or otherwise, able to produce a continuous estimate of VF change, as the time-to-progression is only concerned with the point in time at which deterioration from baseline is reached according to a prespecified cut-off. This might have important implications for incorporating more complex methods, for example, based on artificial intelligence,^50^^,^^51^ to model potential nonlinear behaviors in VF progression. Finally, using a model-based estimate of progression has the advantage of curbing the effect of perimetric noise and avoids “event-reversals,” in which a subject is observed to have progressed at a certain point in time because of test fluctuations only to revert back to values closer to baseline with the following tests. This occurrence can be surprisingly common when progression is evaluated at each time point, ignoring the whole series.^52^ In our clinical sample, the percentage of eyes with “event-reversals” (i.e. change from baseline not sustained in at least one field after the first event) was 48% for the FDA-7 criterion and 65% for the GPA-like criterion.

It should be mentioned that our comparison was performed with standard LMM, which assume a normal distribution of the random effects. Other groups have proposed implementations of LMMs assuming skewed distributions for the random effects,^11^ which may translate in an improved power especially when most of the average difference is found in the negative tail of the distribution. However, on the one hand, these would require sophisticated methodology to be implemented, which would limit immediate widespread adoption and uptake by regulatory bodies. On the other hand, fixed effects in LMMs would still quantify the average difference in RoP and retain the same issues regarding the translation of the results to the risk for individual patients, regardless of the distribution assumed for the random effects. However, a skewed random effect distribution could be used to more accurately estimate the treatment effect on the proportion of fast progressors, either by applying our proposed time-to-event methodology to the random effect estimates or by making inference based on the parameters of the skewed random effect distribution estimated from the study cohort.

Our analysis has limitations. Our anonymized dataset is composed of VF tests collected for clinical practice and without a clear label that identifies patients with glaucoma. Still, it is reasonable to assume that patients with more than 10 VFs over 4 years and with at least 2 fields with an MD < −2 dB would be, at the very least, glaucoma suspects, although the effect of concomitant disease cannot be excluded. Tests were also not collected in a systematic way, meaning that the frequency of testing was relatively sparse when compared to what might happen in an RCT (on average, 1.3 VFs per year). The effect of “event-reversals” clearly affects the definition of our progression labels. However, this analysis was meant to test the power of trend-based models against FDA-compatible criteria, rather than testing their ability to determine true progression. The other assumption in the simulations was that the same linear trend would describe progression for the whole follow-up period. This was done to obtain robust estimates of the assumed “true” RoP for each eye, because many patients (2559, 76%) had < 4 VFs in their first 2 years of follow-up. However, although clinical management might have altered the natural progression of the disease, large deviations from linearity are not expected because this dataset did not include tests performed after incisional glaucoma surgery. Finally, by nature of the simulation method, the two arms of the trial could not be fully randomized because we needed to selectively sample progressed and not progressed patients to simulate the treatment effect. By design, this creates a disparity in the number of patients at higher risk of progressing in the two arms. In fact, this feature was exploited to simulate the desired effect. However, we took care to control for baseline damage during sampling, to make sure that the disease-dependent level of noise was equivalent between the two arms. One important aspect to consider is that simulations likely do not completely reproduce all the nuances of real data. For example, the surprisingly low power of event-based methods in simulated series, despite attempting to detect progression defined according to the same criteria, could be caused by discrepancies between simulations and reality. We provide a supplementary analysis showing that both event-based methods used in our study performed very similarly in the real series and in series with VF tests simulated at the same time points as the real tests. This shows that our results are unlikely to be artificially determined by our simulation methodology.

In conclusion, a time-to-event analysis based on linear regression of the MD offers the same power as LMMs in detecting differences in glaucoma progression labeled with FDA-compatible criteria on long clinical VF series. At the same time, a time-to-event analysis provides a method to define outcomes more clearly for trials and to explore the change in the risk of progression.

## Supplementary Material

Supplement 1
