# Supplementary material for: Validating Trend-Based End Points for Neuroprotection Trials in Glaucoma
Source: Transl Vis Sci Technol. 2023 Oct 31;12(10):20. doi: 10.1167/tvst.12.10.20 (PMC10619697; doi:10.1167/tvst.12.10.20)

Supplementary to: Validating trend-based endpoints for neuroprotection trials in glaucoma

Power

Power for the different methods using the two different FDA-compatible criteria (FDA-7 and GPA-like) to detect progressing eyes. Power is reported as percentage. LMM = Linear Mixed Model. Cells containing estimates whose 95% confidence intervals extend beyond 80% are highlighted in grey.

FDA-7, no effect (false positives)

Sample size	FDA-7	Time-to-progression				LMM
		0.5 dB	1 dB	2 dB	Combined	
N = 100	4.2 [3.0,5.4]	4.0 [2.8,5.2]	3.3 [2.2,4.4]	3.1 [2.0,4.2]	2.3 [1.4,3.2]	3.7 [2.5,4.9]
N = 200	2.9 [1.9,3.9]	2.8 [1.8,3.8]	2.6 [1.6,3.6]	2.8 [1.8,3.8]	2.4 [1.5,3.3]	3.0 [1.9,4.1]
N = 300	4.5 [3.2,5.8]	3.0 [1.9,4.1]	2.9 [1.9,3.9]	4.1 [2.9,5.3]	2.7 [1.7,3.7]	3.3 [2.2,4.4]
N = 400	4.3 [3.0,5.6]	4.4 [3.1,5.7]	3.7 [2.5,4.9]	3.3 [2.2,4.4]	2.3 [1.4,3.2]	3.7 [2.5,4.9]
N = 500	3.3 [2.2,4.4]	2.8 [1.8,3.8]	3.1 [2.0,4.2]	4.1 [2.9,5.3]	3.1 [2.0,4.2]	3.3 [2.2,4.4]
N = 600	3.5 [2.4,4.6]	3.5 [2.4,4.6]	3.0 [1.9,4.1]	4.9 [3.6,6.2]	2.6 [1.6,3.6]	3.4 [2.3,4.5]
N = 700	2.4 [1.5,3.3]	3.0 [1.9,4.1]	3.6 [2.4,4.8]	4.0 [2.8,5.2]	2.5 [1.5,3.5]	3.3 [2.2,4.4]
N = 800	2.8 [1.8,3.8]	3.2 [2.1,4.3]	3.2 [2.1,4.3]	3.7 [2.5,4.9]	2.4 [1.5,3.3]	3.2 [2.1,4.3]
N = 900	3.9 [2.7,5.1]	3.8 [2.6,5.0]	2.8 [1.8,3.8]	4.1 [2.9,5.3]	2.4 [1.5,3.3]	3.0 [1.9,4.1]
N = 1000	3.0 [1.9,4.1]	3.5 [2.4,4.6]	2.8 [1.8,3.8]	4.2 [3.0,5.4]	2.3 [1.4,3.2]	4.2 [3.0,5.4]
N = 1100	4.2 [3.0,5.4]	1.8 [1.0,2.6]	3.6 [2.4,4.8]	4.2 [3.0,5.4]	2.1 [1.2,3.0]	3.0 [1.9,4.1]
N = 1200	3.8 [2.6,5.0]	3.3 [2.2,4.4]	2.6 [1.6,3.6]	3.7 [2.5,4.9]	2.4 [1.5,3.3]	2.6 [1.6,3.6]
N = 1300	3.7 [2.5,4.9]	2.7 [1.7,3.7]	2.0 [1.1,2.9]	3.9 [2.7,5.1]	1.4 [0.7,2.1]	2.9 [1.9,3.9]

FDA-7, power for 20% effect

Sample size	FDA-7	Time-to-progression				LMM
		0.5 dB	1 dB	2 dB	Combined	
N = 100	4.3 [3.0, 5.6]	4.9 [3.6, 6.2]	4.7 [3.4, 6.0]	5.4 [4.0, 6.8]	2.7 [1.7, 3.7]	4.6 [3.3, 5.9]
N = 200	5.9 [4.4, 7.4]	10.7 [8.8,12.6]	10.2 [8.3,12.1]	7.0 [5.4, 8.6]	7.3 [5.7, 8.9]	9.6 [7.8,11.4]
N = 300	5.3 [3.9, 6.7]	13.2 [11.1,15.3]	12.2 [10.2,14.2]	8.9 [7.1,10.7]	9.3 [7.5,11.1]	13.8 [11.7,15.9]
N = 400	6.8 [5.2, 8.4]	13.5 [11.4,15.6]	13.5 [11.4,15.6]	8.7 [7.0,10.4]	11.0 [9.1,12.9]	12.7 [10.6,14.8]
N = 500	6.5 [5.0, 8.0]	20.2 [17.7,22.7]	20.2 [17.7,22.7]	11.1 [9.2,13.0]	15.4 [13.2,17.6]	19.5 [17.0,22.0]
N = 600	8.6 [6.9,10.3]	24.8 [22.1,27.5]	23.7 [21.1,26.3]	16.4 [14.1,18.7]	19.9 [17.4,22.4]	23.7 [21.1,26.3]
N = 700	9.1 [7.3,10.9]	24.8 [22.1,27.5]	24.9 [22.2,27.6]	14.5 [12.3,16.7]	19.7 [17.2,22.2]	23.5 [20.9,26.1]
N = 800	10.1 [8.2,12.0]	31.0 [28.1,33.9]	29.3 [26.5,32.1]	17.3 [15.0,19.6]	25.6 [22.9,28.3]	27.6 [24.8,30.4]
N = 900	11.9 [9.9,13.9]	35.9 [32.9,38.9]	34.1 [31.2,37.0]	21.3 [18.8,23.8]	31.8 [28.9,34.7]	33.6 [30.7,36.5]
N = 1000	11.8 [9.8,13.8]	38.6 [35.6,41.6]	36.6 [33.6,39.6]	22.5 [19.9,25.1]	34.3 [31.4,37.2]	35.5 [32.5,38.5]
N = 1100	12.3 [10.3,14.3]	43.2 [40.1,46.3]	41.8 [38.7,44.9]	22.5 [19.9,25.1]	34.3 [31.4,37.2]	40.8 [37.8,43.8]
N = 1200	12.8 [10.7,14.9]	47.0 [43.9,50.1]	42.3 [39.2,45.4]	22.5 [19.9,25.1]	40.5 [37.5,43.5]	42.1 [39.0,45.2]
N = 1300	13.6 [11.5,15.7]	51.8 [48.7,54.9]	48.5 [45.4,51.6]	28.5 [25.7,31.3]	44.3 [41.2,47.4]	46.9 [43.8,50.0]

FDA-7, power for 30% effect

Sample size	FDA-7	Time-to-progression				LMM
		0.5 dB	1 dB	2 dB	Combined	
**N = 100**	6.2 [4.7, 7.7]	9.6 [7.8,11.4]	8.1 [6.4, 9.8]	6.5 [5.0, 8.0]	6.2 [4.7, 7.7]	8.0 [6.3, 9.7]
**N = 200**	5.2 [3.8, 6.6]	17.4 [15.1,19.7]	15.8 [13.5,18.1]	9.6 [7.8,11.4]	12.3 [10.3,14.3]	16.4 [14.1,18.7]
**N = 300**	8.5 [6.8,10.2]	30.4 [27.5,33.3]	29.4 [26.6,32.2]	15.2 [13.0,17.4]	25.0 [22.3,27.7]	28.4 [25.6,31.2]
**N = 400**	9.2 [7.4,11.0]	34.1 [31.2,37.0]	30.6 [27.7,33.5]	16.1 [13.8,18.4]	28.5 [25.7,31.3]	27.8 [25.0,30.6]
**N = 500**	13.8 [11.7,15.9]	43.2 [40.1,46.3]	41.8 [38.7,44.9]	22.0 [19.4,24.6]	35.8 [32.8,38.8]	39.7 [36.7,42.7]
**N = 600**	12.9 [10.8,15.0]	48.3 [45.2,51.4]	46.8 [43.7,49.9]	26.4 [23.7,29.1]	42.6 [39.5,45.7]	44.5 [41.4,47.6]
**N = 700**	17.2 [14.9,19.5]	58.6 [55.5,61.7]	55.2 [52.1,58.3]	33.2 [30.3,36.1]	52.1 [49.0,55.2]	50.5 [47.4,53.6]
**N = 800**	18.1 [15.7,20.5]	65.2 [62.2,68.2]	61.8 [58.8,64.8]	34.7 [31.7,37.7]	59.0 [56.0,62.0]	59.7 [56.7,62.7]
**N = 900**	21.6 [19.0,24.2]	69.0 [66.1,71.9]	67.3 [64.4,70.2]	39.1 [36.1,42.1]	65.0 [62.0,68.0]	63.7 [60.7,66.7]
**N = 1000**	23.2 [20.6,25.8]	74.7 [72.0,77.4]	74.7 [72.0,77.4]	42.5 [39.4,45.6]	69.6 [66.7,72.5]	70.6 [67.8,73.4]
**N = 1100**	23.7 [21.1,26.3]	78.8 [76.3,81.3]	78.2 [75.6,80.8]	45.9 [42.8,49.0]	76.1 [73.5,78.7]	72.8 [70.0,75.6]
**N = 1200**	27.7 [24.9,30.5]	82.3 [79.9,84.7]	81.3 [78.9,83.7]	53.0 [49.9,56.1]	80.2 [77.7,82.7]	79.5 [77.0,82.0]
**N = 1300**	28.4 [25.6,31.2]	83.6 [81.3,85.9]	83.0 [80.7,85.3]	53.5 [50.4,56.6]	82.2 [79.8,84.6]	81.9 [79.5,84.3]

FDA-7, power for 50% effect

Sample size	FDA-7	Time-to-progression				LMM
		0.5 dB	1 dB	2 dB	Combined	
**N = 100**	7.9 [6.2, 9.6]	23.0 [20.4, 25.6]	24.6 [21.9, 27.3]	13.3 [11.2,15.4]	18.9 [16.5, 21.3]	22.1 [19.5, 24.7]
**N = 200**	12.7 [10.6,14.8]	48.8 [45.7, 51.9]	48.7 [45.6, 51.8]	26.1 [23.4,28.8]	42.7 [39.6, 45.8]	43.6 [40.5, 46.7]
**N = 300**	16.8 [14.5,19.1]	68.3 [65.4, 71.2]	67.4 [64.5, 70.3]	37.3 [34.3,40.3]	62.4 [59.4, 65.4]	62.5 [59.5, 65.5]
**N = 400**	24.0 [21.4,26.6]	78.6 [76.1, 81.1]	76.8 [74.2, 79.4]	47.9 [44.8,51.0]	76.5 [73.9, 79.1]	74.3 [71.6, 77.0]
**N = 500**	30.1 [27.3,32.9]	89.4 [87.5, 91.3]	88.3 [86.3, 90.3]	59.1 [56.1,62.1]	87.2 [85.1, 89.3]	85.3 [83.1, 87.5]
**N = 600**	34.1 [31.2,37.0]	92.5 [90.9, 94.1]	92.9 [91.3, 94.5]	67.6 [64.7,70.5]	92.0 [90.3, 93.7]	91.0 [89.2, 92.8]
**N = 700**	40.2 [37.2,43.2]	95.7 [94.4, 97.0]	95.2 [93.9, 96.5]	72.4 [69.6,75.2]	95.4 [94.1, 96.7]	94.2 [92.8, 95.6]
**N = 800**	48.5 [45.4,51.6]	98.6 [97.9, 99.3]	99.1 [98.5, 99.7]	83.9 [81.6,86.2]	98.3 [97.5, 99.1]	97.4 [96.4, 98.4]
**N = 900**	45.5 [42.4,48.6]	98.6 [97.9, 99.3]	99.1 [98.5, 99.7]	85.6 [83.4,87.8]	98.8 [98.1, 99.5]	98.6 [97.9, 99.3]
**N = 1000**	55.3 [52.2,58.4]	99.6 [99.2,100.0]	99.9 [99.7,100.1]	89.2 [87.3,91.1]	99.7 [99.4,100.0]	99.2 [98.6, 99.8]
**N = 1100**	60.8 [57.8,63.8]	99.9 [99.7,100.1]	99.8 [99.5,100.1]	91.7 [90.0,93.4]	99.8 [99.5,100.1]	99.5 [99.1, 99.9]
**N = 1200**	62.0 [59.0,65.0]	99.7 [99.4,100.0]	99.8 [99.5,100.1]	92.9 [91.3,94.5]	100.0 [100.0,100.0]	100.0 [100.0,100.0]
**N = 1300**						

GPA-like, no effect (false positives)

Sample size	GPA-like	Time-to-progression 0.5 dB	1 dB	2 dB	Combined	LMM
**N = 100**	4.7 [3.4,3.4]	5.2 [3.8,3.8]	6.2 [4.7,4.7]	3.7 [2.5,2.5]	3.0 [1.9,1.9]	4.4 [3.1,3.1]
**N = 200**	3.4 [2.3,2.3]	3.9 [2.7,2.7]	3.7 [2.5,2.5]	4.1 [2.9,2.9]	2.2 [1.3,1.3]	4.1 [2.9,2.9]
**N = 300**	4.1 [2.9,2.9]	4.0 [2.8,2.8]	3.8 [2.6,2.6]	4.0 [2.8,2.8]	2.4 [1.5,1.5]	4.6 [3.3,3.3]
**N = 400**	3.9 [2.7,2.7]	3.2 [2.1,2.1]	4.3 [3.0,3.0]	4.8 [3.5,3.5]	2.5 [1.5,1.5]	3.3 [2.2,2.2]
**N = 500**	4.8 [3.5,3.5]	3.5 [2.4,2.4]	4.6 [3.3,3.3]	4.3 [3.0,3.0]	3.4 [2.3,2.3]	3.9 [2.7,2.7]
**N = 600**	4.0 [2.8,2.8]	4.3 [3.0,3.0]	4.4 [3.1,3.1]	4.2 [3.0,3.0]	3.7 [2.5,2.5]	3.4 [2.3,2.3]
**N = 700**	4.1 [2.9,2.9]	4.5 [3.2,3.2]	4.1 [2.9,2.9]	4.8 [3.5,3.5]	3.7 [2.5,2.5]	3.3 [2.2,2.2]
**N = 800**	3.1 [2.0,2.0]	3.6 [2.4,2.4]	4.9 [3.6,3.6]	3.5 [2.4,2.4]	2.7 [1.7,1.7]	3.5 [2.4,2.4]
**N = 900**	4.0 [2.8,2.8]	4.6 [3.3,3.3]	4.2 [3.0,3.0]	4.4 [3.1,3.1]	2.7 [1.7,1.7]	3.9 [2.7,2.7]
**N = 1000**	5.1 [3.7,3.7]	3.2 [2.1,2.1]	4.0 [2.8,2.8]	4.2 [3.0,3.0]	2.5 [1.5,1.5]	3.4 [2.3,2.3]
**N = 1100**	4.7 [3.4,3.4]	4.6 [3.3,3.3]	3.4 [2.3,2.3]	3.5 [2.4,2.4]	3.1 [2.0,2.0]	3.2 [2.1,2.1]
**N = 1200**	4.0 [2.8,2.8]	3.6 [2.4,2.4]	3.6 [2.4,2.4]	4.7 [3.4,3.4]	2.9 [1.9,1.9]	4.0 [2.8,2.8]
**N = 1300**	3.9 [2.7,2.7]	3.3 [2.2,2.2]	3.7 [2.5,2.5]	3.9 [2.7,2.7]	3.1 [2.0,2.0]	3.5 [2.4,2.4]

GPA-like, power for 20% effect

Sample size	GPA-like	Time-to-progression 0.5 dB	1 dB	2 dB	Combined	LMM
**N = 100**	4.0 [2.8, 5.2]	7.8 [6.1, 9.5]	7.6 [6.0, 9.2]	5.0 [3.6, 6.4]	5.2 [3.8, 6.6]	5.5 [4.1, 6.9]
**N = 200**	7.0 [5.4, 8.6]	8.8 [7.0,10.6]	8.3 [6.6,10.0]	5.6 [4.2, 7.0]	6.3 [4.8, 7.8]	7.2 [5.6, 8.8]
**N = 300**	6.2 [4.7, 7.7]	12.3 [10.3,14.3]	11.6 [9.6,13.6]	7.4 [5.8, 9.0]	9.7 [7.9,11.5]	13.2 [11.1,15.3]
**N = 400**	8.4 [6.7,10.1]	14.5 [12.3,16.7]	11.7 [9.7,13.7]	9.5 [7.7,11.3]	11.3 [9.3,13.3]	13.6 [11.5,15.7]
**N = 500**	10.3 [8.4,12.2]	19.7 [17.2,22.2]	17.8 [15.4,20.2]	12.6 [10.5,14.7]	14.5 [12.3,16.7]	17.2 [14.9,19.5]
**N = 600**	9.5 [7.7,11.3]	20.9 [18.4,23.4]	19.9 [17.4,22.4]	12.5 [10.5,14.5]	17.1 [14.8,19.4]	20.5 [18.0,23.0]
**N = 700**	11.8 [9.8,13.8]	25.1 [22.4,27.8]	22.0 [19.4,24.6]	11.3 [9.3,13.3]	19.8 [17.3,22.3]	20.9 [18.4,23.4]
**N = 800**	14.1 [11.9,16.3]	28.3 [25.5,31.1]	26.9 [24.2,29.6]	14.2 [12.0,16.4]	23.0 [20.4,25.6]	26.1 [23.4,28.8]
**N = 900**	13.5 [11.4,15.6]	27.4 [24.6,30.2]	26.6 [23.9,29.3]	16.2 [13.9,18.5]	22.3 [19.7,24.9]	25.0 [22.3,27.7]
**N = 1000**	15.9 [13.6,18.2]	34.0 [31.1,36.9]	31.9 [29.0,34.8]	19.1 [16.7,21.5]	28.1 [25.3,30.9]	30.0 [27.2,32.8]
**N = 1100**	16.0 [13.7,18.3]	32.9 [30.0,35.8]	32.8 [29.9,35.7]	18.7 [16.3,21.1]	28.5 [25.7,31.3]	33.7 [30.8,36.6]
**N = 1200**	18.6 [16.2,21.0]	39.3 [36.3,42.3]	36.2 [33.2,39.2]	21.7 [19.1,24.3]	31.9 [29.0,34.8]	36.8 [33.8,39.8]
**N = 1300**	19.6 [17.1,22.1]	42.8 [39.7,45.9]	41.2 [38.1,44.3]	22.5 [19.9,25.1]	36.5 [33.5,39.5]	37.7 [34.7,40.7]

GPA-like, power for 30% effect

Sample size	GPA-like	Time-to-progression 0.5 dB	Time-to-progression 1 dB	Time-to-progression 2 dB	Time-to-progression Combined	LMM
**N = 100**	6.0 [4.5, 7.5]	10.1 [8.2,12.0]	9.5 [7.7,11.3]	6.0 [4.5, 7.5]	6.2 [4.7, 7.7]	10.4 [8.5,12.3]
**N = 200**	8.3 [6.6,10.0]	15.9 [13.6,18.2]	15.7 [13.4,18.0]	9.7 [7.9,11.5]	12.7 [10.6,14.8]	15.3 [13.1,17.5]
**N = 300**	13.7 [11.6,15.8]	21.0 [18.5,23.5]	20.9 [18.4,23.4]	12.7 [10.6,14.8]	16.4 [14.1,18.7]	21.3 [18.8,23.8]
**N = 400**	14.6 [12.4,16.8]	32.0 [29.1,34.9]	31.4 [28.5,34.3]	17.7 [15.3,20.1]	25.3 [22.6,28.0]	28.8 [26.0,31.6]
**N = 500**	17.0 [14.7,19.3]	37.3 [34.3,40.3]	35.6 [32.6,38.6]	19.1 [16.7,21.5]	29.7 [26.9,32.5]	32.0 [29.1,34.9]
**N = 600**	19.1 [16.7,21.5]	45.3 [42.2,48.4]	41.9 [38.8,45.0]	23.2 [20.6,25.8]	39.1 [36.1,42.1]	40.8 [37.8,43.8]
**N = 700**	23.3 [20.7,25.9]	49.6 [46.5,52.7]	47.0 [43.9,50.1]	25.0 [22.3,27.7]	42.2 [39.1,45.3]	45.7 [42.6,48.8]
**N = 800**	27.4 [24.6,30.2]	54.5 [51.4,57.6]	51.6 [48.5,54.7]	29.7 [26.9,32.5]	48.8 [45.7,51.9]	51.8 [48.7,54.9]
**N = 900**	29.6 [26.8,32.4]	57.6 [54.5,60.7]	54.6 [51.5,57.7]	31.5 [28.6,34.4]	51.6 [48.5,54.7]	51.8 [48.7,54.9]
**N = 1000**	32.3 [29.4,35.2]	66.6 [63.7,69.5]	62.3 [59.3,65.3]	34.7 [31.7,37.7]	62.1 [59.1,65.1]	63.2 [60.2,66.2]
**N = 1100**	33.0 [30.1,35.9]	71.0 [68.2,73.8]	68.8 [65.9,71.7]	42.2 [39.1,45.3]	64.8 [61.8,67.8]	65.6 [62.7,68.5]
**N = 1200**	38.0 [35.0,41.0]	74.5 [71.8,77.2]	74.0 [71.3,76.7]	46.2 [43.1,49.3]	71.5 [68.7,74.3]	71.1 [68.3,73.9]
**N = 1300**						

GPA-like, power for 50% effect

Sample size	GPA-like	Time-to-progression 0.5 dB	Time-to-progression 1 dB	Time-to-progression 2 dB	Time-to-progression Combined	LMM
**N = 100**	12.6 [10.5,14.7]	26.7 [24.0,29.4]	21.6 [19.0,24.2]	11.9 [9.9,13.9]	18.2 [15.8,20.6]	25.1 [22.4,27.8]
**N = 200**	19.1 [16.7,21.5]	41.5 [38.4,44.6]	42.9 [39.8,46.0]	23.1 [20.5,25.7]	36.8 [33.8,39.8]	40.8 [37.8,43.8]
**N = 300**	27.7 [24.9,30.5]	55.8 [52.7,58.9]	54.0 [50.9,57.1]	31.9 [29.0,34.8]	51.7 [48.6,54.8]	54.3 [51.2,57.4]
**N = 400**	34.3 [31.4,37.2]	71.9 [69.1,74.7]	68.5 [65.6,71.4]	41.9 [38.8,45.0]	68.1 [65.2,71.0]	68.7 [65.8,71.6]
**N = 500**	41.6 [38.5,44.7]	80.3 [77.8,82.8]	80.2 [77.7,82.7]	49.9 [46.8,53.0]	78.0 [75.4,80.6]	76.3 [73.7,78.9]
**N = 600**	50.3 [47.2,53.4]	85.9 [83.7,88.1]	83.4 [81.1,85.7]	58.2 [55.1,61.3]	83.4 [81.1,85.7]	81.9 [79.5,84.3]
**N = 700**	58.4 [55.3,61.5]	92.0 [90.3,93.7]	90.7 [88.9,92.5]	65.7 [62.8,68.6]	89.9 [88.0,91.8]	87.7 [85.7,89.7]
**N = 800**	64.9 [61.9,67.9]	95.6 [94.3,96.9]	94.1 [92.6,95.6]	74.5 [71.8,77.2]	94.0 [92.5,95.5]	93.4 [91.9,94.9]
**N = 900**	69.1 [66.2,72.0]	96.0 [94.8,97.2]	96.9 [95.8,98.0]	75.1 [72.4,77.8]	96.0 [94.8,97.2]	95.3 [94.0,96.6]
**N = 1000**	76.1 [73.5,78.7]	98.7 [98.0,99.4]	98.1 [97.3,98.9]	83.4 [81.1,85.7]	97.9 [97.0,98.8]	97.9 [97.0,98.8]
**N = 1100**						
**N = 1200**						
**N = 1300**						

Percentage change in the average rate of progression

Average simulated change in the rate of progression (RoP) slope for different simulated effect, for all the simulated trials. The percentage change in slope is calculated as $(RoP_{Placebo} - RoP_{Treatment})/RoP_{Placebo}$. The diagonal solid line indicates equivalence. N = sample size per arm.

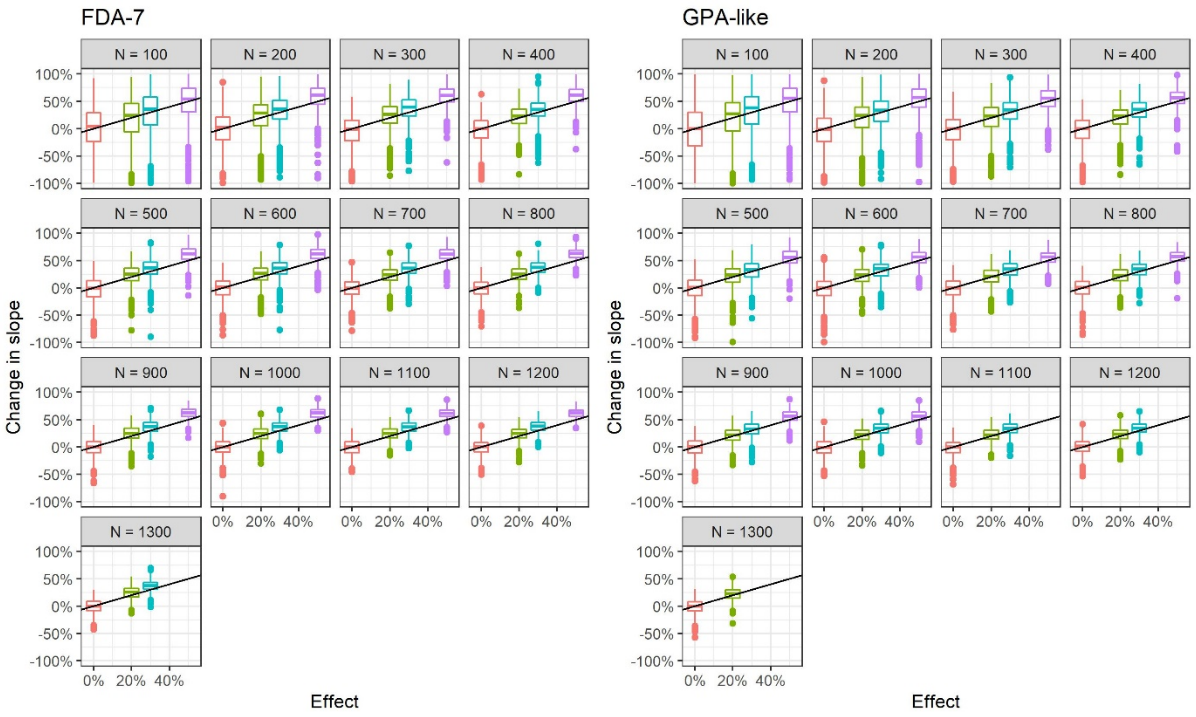

Event-based progression in simulated series

The graphs show survival curves for the two event-based progression criteria used in this study applied to simulated series with tests at the same time-points as the original series, stratified by level of damage. The survival curves are similar **Figure 3**, with some discrepancies for advanced patients with the GPA-like criteria. Censored data marks are omitted for clarity.

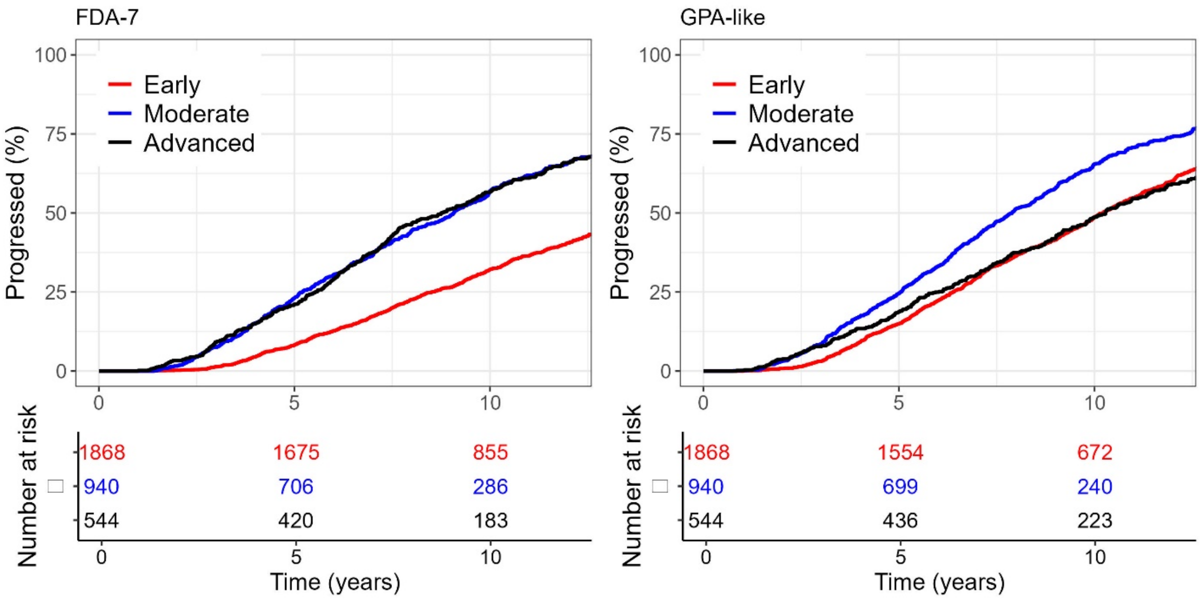

Power curves excluding patients with early damage

Power curves for different treatment effect sizes for all the proposed methods, using the two FDA-compatible criteria for classification (either GPA-like or FDA-7, according to which was used for the labelling of progressing eyes in the original clinical series). The error bars indicate the 95%-Confidence Intervals. These curves are produced by excluding patient with early visual field damage (Mean Deviation > -6 dB). The curves are presented on the same scale as Figure 4. The smaller range reflects the smaller maximum sample size available by including only patients with intermediate and advanced eyes.

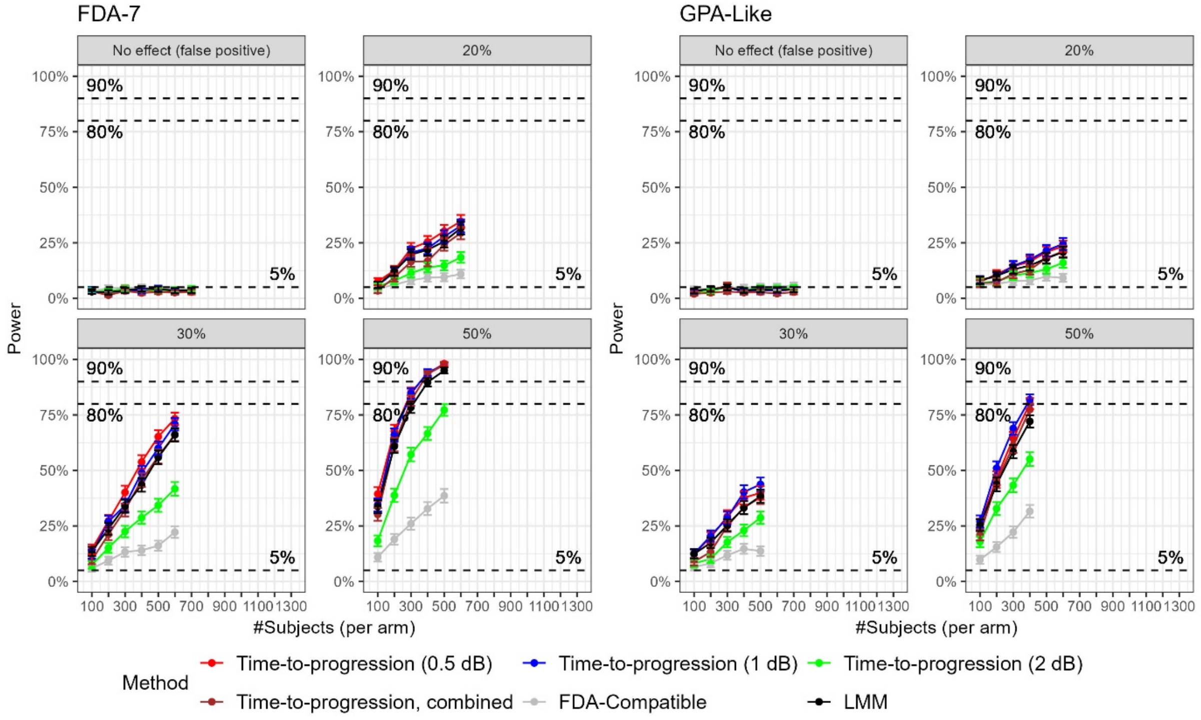

Supplement: Supplement 1 [file tvst-12-10-20_s001.pdf]
